# The United States Chronic Thromboembolic Pulmonary Hypertension Registry: Protocol for a Prospective, Longitudinal Study

**DOI:** 10.2196/25397

**Published:** 2021-05-25

**Authors:** Kim M Kerr, C Greg Elliott, Raymond L Benza, Richard N Channick, Kelly M Chin, R Duane Davis, Sonia Jain, Andrea Z LaCroix, Michael M Madani, Vallerie V McLaughlin, Myung H Park, Victor F Tapson, William R Auger

**Affiliations:** 1 Division of Pulmonary, Critical Care & Sleep Medicine University of California San Diego La Jolla, CA United States; 2 Department of Medicine Intermountain Medical Center Murray, UT United States; 3 Division of Cardiovascular Medicine Ohio State University Columbus, OH United States; 4 Division of Pulmonary and Critical Care Medicine University of California Los Angeles Los Angeles, CA United States; 5 Division of Pulmonary and Critical Care University of Texas Southwestern Dallas, TX United States; 6 Thoracic and Cardiac Surgery AdventHealth Transplant Institute Orlando, FL United States; 7 Division of Biostatistics and Bioinformatics University of California San Diego La Jolla, CA United States; 8 Division of Epidemiology University of California San Diego La Jolla, CA United States; 9 Division of Cardiovascular and Thoracic Surgery University of California San Diego La Jolla, CA United States; 10 Division of Cardiovascular Medicine University of Michigan Ann Arbor, MI United States; 11 Department of Cardiology Virginia Mason Franciscan Health Tacoma, WA United States; 12 Division of Pulmonary and Critical Care Medicine Cedars-Sinai Medical Center Los Angeles, CA United States; 13 Division of Cardiology Temple University Philadelphia, PA United States

**Keywords:** CTEPH, pulmonary hypertension, pulmonary embolism, registry, surgical, nonsurgical, therapy, treatment

## Abstract

**Background:**

Chronic thromboembolic pulmonary hypertension (CTEPH) is a rare sequela of acute pulmonary embolism that is treatable when recognized. Awareness of this disease has increased with recent advancements in therapeutic options, but delays in diagnosis remain common, and diagnostic and treatment guidelines are often not followed. Data gathered from international registries have improved our understanding of CTEPH, but these data may not be applicable to the US population owing to differences in demographics and medical practice patterns.

**Objective:**

The US CTEPH Registry (US-CTEPH-R) was developed to provide essential information to better understand the demographics, risk factors, evaluation, and treatment of CTEPH in the United States, as well as the short- and long-term outcomes of surgical and nonsurgical therapies in the modern treatment era.

**Methods:**

Thirty sites throughout the United States enrolled 750 subjects in this prospective, longitudinal, observational registry of patients newly diagnosed with CTEPH. Enrollment criteria included a mean pulmonary artery pressure ≥25 mmHg by right heart catheterization and radiologic confirmation of CTEPH by a multidisciplinary adjudication committee. Following enrollment, subjects were followed biannually until the conclusion of the study. Quality of life surveys were administered at enrollment and biannually, and all other testing was at the discretion of the treating clinician. Details regarding surgical therapy, balloon pulmonary angioplasty, and medical therapy were collected at enrollment and at follow-up, as well as information related to health care utilization and survival.

**Results:**

Data from this registry will improve understanding of the demographics, risk factors, and treatment patterns of patients with CTEPH, and the longitudinal impact of therapies on quality of life, health care utilization, and survival.

**Conclusions:**

This manuscript details the methodology and design of the first large, prospective, longitudinal registry of patients with CTEPH in the United States.

**Trial Registration:**

ClinicalTrials.gov NCT02429284; https://www.clinicaltrials.gov/ct2/show/NCT02429284

**International Registered Report Identifier (IRRID):**

DERR1-10.2196/25397

## Introduction

Chronic thromboembolic pulmonary hypertension (CTEPH) is a subset of pulmonary hypertension (PH) characterized by obstruction of the pulmonary arteries with fibrotic material and vascular remodeling, which leads to increased pulmonary arterial pressure and right ventricular failure. Although clinical presentation and the pathological changes of the pulmonary vasculature share some characteristics with pulmonary arterial hypertension (PAH), the etiology, diagnosis, and treatment of CTEPH are quite distinct from those of PAH [1]. Registries that focus on PAH have been established [2-6]; however, there has never been an organized collection of multicenter CTEPH data in the United States.

The diagnostic and therapeutic landscape for CTEPH in the United States has changed dramatically in recent years. Digital subtraction pulmonary angiography (DSA) was long considered the gold standard for the diagnosis of CTEPH and determination of operability; however, technological improvements in computerized axial tomography angiography (CTA) have replaced invasive pulmonary angiography in many centers, and magnetic resonance imaging is used at some centers for both angiography (MRA) and assessment of right ventricular function. Nuclear ventilation perfusion scanning remains the recommended screening modality for CTEPH, although dual-energy computed tomography is emerging as a technology that provides the combined imaging required (perfusion mapping and pulmonary angiography) for the evaluation of CTEPH [7,8].

Multiple new treatment options have recently become available to patients with CTEPH, particularly those with inoperable disease or residual PH after surgery. Advances in pulmonary thromboendarterectomy (PTE) techniques have changed the definition of operability, selecting patients with more distal segmental and subsegmental disease who previously were classified to have inoperable disease and are now deemed as surgical candidates. Balloon pulmonary angioplasty (BPA) is now a therapeutic option for those who are not surgical candidates or patients with residual PH following PTE. Riociguat, the first Food and Drug Administration–approved drug for the treatment of CTEPH, is also an option for this subpopulation [9,10].

Despite a growing literature, substantial knowledge gaps remain regarding our understanding of CTEPH. A CTEPH registry involving 26 centers from Europe and one from Canada reported valuable data elucidating the epidemiology, risk factors, and outcomes in this mostly European cohort of patients newly diagnosed with CTEPH [11,12]. However, whether these data are applicable to the US population remains unclear given demographic and cultural differences, as well as the regional disparities in the practice of medicine between Europe and the United States. In addition, this European registry was established prior to the availability of BPA and riociguat for the treatment of CTEPH.

The dramatic changes in the diagnosis and management of CTEPH, and the accompanying knowledge gaps motivated us to organize the first US CTEPH registry. The United States Chronic Thromboembolic Pulmonary Hypertension Registry (US-CTEPH-R) is a contemporary CTEPH registry that will provide data for patients with CTEPH diagnosed between 2014 and 2018. Patient demographics, medical history, symptoms, timeline to diagnosis, risk factors, diagnostic approach, disease management, and long-term outcomes postintervention have been collected. Among the most important issues we sought to address are the longitudinal data on quality of life and health care utilization, and discrepancies between identified subgroups such as gender and race.

## Methods

### US-CTEPH-R Objectives

The US-CTEPH-R has been established under the guidance of a multidisciplinary Steering Committee consisting of physicians, surgeons, and scientists with expertise in the diagnosis and management of CTEPH and related diseases. The mission of the Registry is to promote a greater understanding of the epidemiology, pathophysiology, evaluation, treatment, and outcomes of patients with CTEPH through shared information, education, and collaborative investigation among PH centers throughout the United States. The Steering Committee defined five overall objectives for the US-CTEPH-R: (1) to characterize the demographics, evaluation, and clinical course of CTEPH; (2) to chronicle short- and long-term outcomes of PTE in patients with operable CTEPH; (3) to evaluate the short- and long-term outcomes of nonsurgically treated CTEPH; (4) to identify pre-, intra-, and postoperative predictors of a successful PTE; and (5) to collect pertinent data that will contribute to the understanding of a successful treatment approach for patients with CTEPH.

The development of the US-CTEPH-R will be an important element in the advancement of understanding CTEPH and improvement in the care of patients who suffer from this debilitating disease.

### Design

The US-CTEPH-R is a multicenter, prospective, longitudinal registry of patients newly diagnosed (within the previous 6 months) with CTEPH. The University of California San Diego (UCSD) is the sponsor and coordinating institution for the study, which was approved by the UCSD Human Research Protection Program (Project #141379). Thirty sites in the United States were selected to participate in the Registry based on a feasibility survey and geographic distribution; all sites obtained permission from their respective institutional review boards. All data were entered into a secure web-based data management system. After providing written informed consent, each patient was assigned a unique numerical patient identifier to maintain confidentiality as required by the Health Insurance Portability and Accountability Act. The first patient was enrolled in April 2015 and the target enrollment of 750 patients was met in March 2018. All subjects were followed biannually until the last subject completed 1-year follow-up in March 2019. Subjects could voluntarily withdraw at any time during the study at which point no additional data were collected.

### Subjects

All consecutive patients diagnosed with CTEPH within 6 months of study consent meeting the inclusion criteria outlined in Textbox 1 were offered participation in the study. The time of diagnosis was defined as the date the final of all three hemodynamic and radiologic entry criteria for CTEPH (right heart catheterization, ventilation perfusion scan, and confirmatory angiography) were met. Exclusion criteria are also listed in Textbox 1. Because this is a US CTEPH registry, only patients who were permanent residents of the United States were included. There were no limitations based on age, use of PH targeted therapy, or excessive pulmonary vascular resistance.

**Inclusion and exclusion criteria for enrollment.**

**Inclusion criteria**

- Patient must be a permanent resident of the United States
- Documentation of the following hemodynamic parameters by right heart catheterization
  Mean pulmonary arterial pressure ≥25 mmHg at rest and
  Pulmonary artery wedge pressure (PAWP) ≤15 mmHg (or >15 mmHg if justified; ie, if the principal investigator felt the etiology of the pulmonary hypertension was due to chronic thromboembolic disease and not postcapillary pulmonary hypertension).
- Radiologic confirmation that chronic thromboembolic disease is the cause of the pulmonary hypertension by
  One or more mismatched perfusion defect(s) by lung ventilation/perfusion scan, and
  Confirmation of chronic thromboembolic disease by evidence of bands/webs, vessel narrowing, or occlusion seen on digital subtraction angiography (DSA), computed tomography pulmonary angiogram (CTA), or magnetic resonance angiography (MRA).
  All subjects must have the radiologic diagnosis of chronic thromboembolic pulmonary hypertension (CTEPH) confirmed by the Adjudication Committee.
- Patients must be diagnosed with chronic thromboembolic pulmonary hypertension within 6 months of being considered for study eligibility (signing of consent to participate). The date of diagnosis will be defined as the time that both hemodynamic criteria have been met and chronic thromboembolic disease is confirmed to be the cause of the pulmonary hypertension by an abnormal ventilation perfusion scan and the presence of chronic thromboembolic disease on CTA, MRA, or DSA. Hemodynamic and radiologic criteria can be met at separate time points; the most recently met criteria time point will be defined as the date of diagnosis.

**Exclusion criteria**

- Patients unwilling or unable to provide written consent for participation in the study. Appropriate surrogate consent will be obtained for pediatric patients as defined by each investigational site’s institutional review board.
- Patients with an underlying medical disorder with an anticipated life expectancy less than 2 years.
- Patients who do not meet inclusion criteria:
  Have not had documentation of hemodynamic criteria by right heart catheterization as outlined in the inclusion criteria
  Do not have radiologic confirmation of chronic thromboembolic disease as outlined in the inclusion criteria
- Patients who have undergone a prior pulmonary thromboendarterectomy surgery or balloon pulmonary angioplasty procedure.
- Meet the criteria for inclusion into Pulmonary Hypertension World Health Organization Groups I, II, III, or V.

### Adjudication

Radiologic imaging was forwarded to the Adjudication Committee using a web-based platform for review. A ventilation perfusion scan and at least one form of pulmonary angiography (CTA, MRA, or DSA) were submitted. Six physicians from multiple centers with extensive clinical experience in CTEPH served on the Adjudication Committee. Successful radiologic adjudication of CTEPH required two independent adjudicators to agree that the imaging met the predefined criteria listed in Textbox 2 [13-16]. In the event the adjudicators did not agree on the diagnosis, a third adjudicator rendered an independent decision as to whether the imaging supported the diagnosis of CTEPH. Two of the three adjudicators had to agree on the diagnosis for the subject to be enrolled in the study and at least one of the adjudicators confirming CTEPH had to come from outside the enrolling institution. When the diagnosis was uncertain based on the submitted imaging, additional clinical history or available imaging could be requested by the Adjudication Committee. Subjects that were deemed to have imaging compatible with acute thromboembolic disease were not entered into the registry unless stability of the findings was documented after 3 months of anticoagulation. Subjects with radiologic imaging supporting the diagnosis of CTEPH were subsequently enrolled and followed longitudinally.

**Chronic thromboembolic pulmonary hypertension (CTEPH) adjudication criteria.**

Diagnosis of CTEPH requires both unmatched perfusion defects on ventilation perfusion scan *and* confirmatory evidence of chronic thromboembolic disease on computed tomography (CT), magnetic resonance (MR), or digital subtraction angiography (DSA).

**CT and MR findings compatible with chronic thromboembolic disease:**

- Eccentric lining material (clot) without distortion of vessels
- Occlusive/recanalized/intraluminal material, associated with vessel distortion and/or narrowing; postobstructive vessel attenuation or “absence” to accompanying occlusive clot
- Intraluminal webs, with or without vessel distortion
- Vessel nonopacification with intimal thickening at vessel origin

**CT and MR findings supporting the diagnosis of CTEPH:**

- Enlarged central pulmonary arteries
- Systemic collateral vessels
- Enlargement of the right atrium/right ventricle
- Mosaic parenchymal perfusion
- Absence of parenchymal findings that can be associated with vessel destruction/poor perfusion (coexisting “stasis” clot)
- Intact large pulmonary veins
- No significant mediastinal pathology

**DSA findings compatible with chronic thromboembolic disease:**

- “Pouching” resulting in partial or total occlusion of the vessel
- Pulmonary arterial webs or bands
- Intimal irregularities
- Abrupt narrowing of a major pulmonary artery
- Obstruction of vessels at their point of origin

Patients with radiographic evidence compatible with acute thromboembolic disease will not be included in the registry unless stability of these findings are documented after 3 months of anticoagulation.

**CT and MR findings suggesting the diagnosis of acute pulmonary embolism:**

- Arterial occlusion with failure to enhance the entire lumen due to a large filling defect
- A partial filling defect surrounded by contrast material
- The “railway track sign,” thromboembolic masses seen floating freely in the lumen, allowing the flow of blood between the wall of the vessel and the embolus
- A peripheral intraluminal filling defect that forms acute angles with the arterial wall

### Data Collection

Data collected at enrollment included demographics, medical history, risk factors for venous thromboembolism and CTEPH, duration of symptoms, time from symptom onset to correct diagnosis, alternative diagnoses initially rendered, specialties of physicians involved in the patient’s care, World Health Organization (WHO) functional class, Short-Form 36 Questionnaire (SF-36v2) and emPHasis-10 [17] scores, as well as biomarkers, 6 minute walk distance, echocardiography, pulmonary function tests, radiologic imaging, hemodynamics, medical therapies (anticoagulants, PH targeted therapies, diuretics, supplemental oxygen, inferior vena cava filters), and determination of operability status.

Following enrollment, the only requirement for participation in the registry was the administration of the health-related quality of life (HRQoL) SF-36v2 and emPHasis-10 questionnaires. All other evaluations and procedures were at the discretion of the investigator.

For subjects undergoing PTE surgery, the following information was collected: date and location of PTE surgery, surgical classification of thrombus, cardiopulmonary bypass, aortic cross clamp and circulatory arrest times, additional procedures performed at the time of PTE, postoperative hemodynamics, length of mechanical ventilation, inotropic support, intensive care unit and hospital length of stay, postoperative complications, mortality, and cause of death when applicable. Therapy at the time of discharge (anticoagulant, inferior vena cava filter, PH targeted therapies, antiarrhythmic, supplemental oxygen) was also recorded.

Subjects who did not undergo PTE surgery had the reason documented and were followed longitudinally. Patients treated with BPA had each session recorded with identification of segments treated, complications, length of stay, radiation exposure, and hemodynamics.

Longitudinal data were collected biannually (every 6 months) on all patients during patient clinic visits, or by patient phone call and/or chart abstraction. Data collected in follow-up include WHO Functional Class, SF-36v2, emPHasis-10, results of follow-up testing (labs, echocardiography, right heart catheterization, radiologic imaging), performance of PTE surgery, BPA, transplant, changes in medications, hospitalizations/emergency room visits, and death.

### Data Analysis

The sample size (750 patients) and duration of follow-up were selected based on estimation of disease incidence from a feasibility survey of US PH centers and budgetary constraints. Aggregate data from all 30 sites will be published, but site-specific data will be confidentially provided to each participating research center. Descriptive statistics will be used to describe demographics, risk factors, time to appropriate testing and diagnosis, PAH targeted medication use, functional status, and hemodynamics at the time of enrollment. Demographic and baseline characteristics will be summarized descriptively. For descriptive comparisons, the Fisher exact test will be used for categorical variables and *t* tests will be used for continuous variables. Appropriate nonparametric alternatives such as the Wilcoxon rank-sum test will be considered if parametric assumptions fail.

Results will be reported as point estimates (odds or hazard ratios, or mean differences across groups, as appropriate) and interval estimates (95% CIs). All tests of significance will be two-sided. A *P* value of .05 or less will be considered statistically significant. Statistical analysis will be performed using the statistical software R 3.5.0.

Descriptive statistics of the perioperative data collected will be presented along with any observations of differences in perioperative practice. Comparisons of hemodynamics, functional status, medication and supplemental oxygen use, HRQoL, and health care utilization will be made between the preoperative data and longitudinal postoperative data.

Descriptive statistics will be used to describe why patients are not operative candidates and the nonsurgical therapies used for treatment. Longitudinal changes in hemodynamics, functional class, HRQoL, medication use, health care utilization, and death will be analyzed using repeated-measures analysis such as mixed models repeated measures. Comparisons of treatment modalities (surgical and nonsurgical) on clinical outcomes will be assessed using regression techniques adjusting for potential confounding variables. Subgroup (such as gender or race) comparisons of risk factors, therapies, and outcomes will be considered, as appropriate. Survival of patients will be analyzed using time-to-event analyses and descriptively summarized using Kaplan-Meier curves.

A separate statistical analysis plan will be developed for each specific investigation prior to statistical analysis.

## Results

Data from this registry will help us better understand the demographics, presentation, and risk factors of the disease, as well as the diagnostic approach and treatment patterns in the United States. Impediments to timely diagnosis or effective therapy may also be identified, providing opportunities to improve health care for patients with CTEPH. Longitudinal data will provide insight into the effect of PTE, BPA, and pharmacologic therapies on health care utilization, HRQoL, and survival, and will contribute to our understanding of optimal treatment strategies for these patients. Comparisons between subgroups such as gender and race may identify disparities in risk factors, presentation, evaluation, treatment, and outcome.

## Discussion

### Overview of Protocol Design

This is the first large, multicenter, observational, longitudinal registry of patients with CTEPH in the United States. A previously published and often quoted European prospective registry that enrolled 679 adults newly diagnosed (≤6 months) with CTEPH found that 36.4% of cases were inoperable in the group of patients who did not undergo PTE surgery experiencing decreased survival [5,6]. However, this study was performed before regulatory approval of riociguat or availability of BPA for the treatment of inoperable CTEPH or residual PH after PTE. Aside from the current availability of these nonsurgical therapies, this US CTEPH registry differs from the previous European registry in several important ways. First, the demographics will be reflective of the US CTEPH patient population with greater racial diversity and differences in comorbidities such as obesity, diabetes, and sleep disordered breathing. Second, pediatric patients are included in this registry. Third, all patients have been adjudicated by a team of CTEPH experts to confirm the diagnosis of CTEPH prior to enrollment. Fourth, outcomes from the patient’s perspective (HRQoL and functional status) will be measured. Finally, patients on PH targeted therapy and undergoing BPA are included.

### Limitations

There are several recognized limitations to this registry. Because it is observational, there is no mandated testing except for obtaining the SF-36v2 and emPHasis-10 responses biannually. Hence, timing of follow-up testing will be different between patients and there may be no follow-up testing on others. Although we are attempting to understand the diagnosis and treatment of CTEPH in the United States, the members of the US-CTEPH-R sites are based at academic PH centers, many offering more advanced PAH and CTEPH therapies. Hence, the population enrolled in this registry will not reflect the general population of patients with CTEPH cared for in the community that are never referred to a PH center. This registry was also performed prior to the change in the definition of PH from a mean pulmonary artery pressure ≥25 mmHg to >20 mmHg by the World Symposium on Pulmonary Hypertension in 2018. In addition, the important group of patients with symptomatic chronic thromboembolic disease without resting PH are not included in this registry.

### Ethical Concerns

As with all clinical research, there is a potential for loss of privacy. To mitigate this risk, each patient was assigned a unique identifying number upon enrollment in the registry, and only the enrolling centers have access to their own specific patient-identifying data. Data were entered into a secure web-based management system and are password-protected. With approval of the Steering Committee, collaborating researchers requesting to analyze portions of the registry data will be given sets of records stripped of all personally identifiable information. In addition, although the US-CTEPH-R was funded by industry (see Acknowledgments), all registry activities are overseen by the Steering Committee. The sponsor had no role in the design of the study, collection and analysis of data, or preparation of manuscripts.

### Conclusion

The US-CTEPH-R with 750 newly diagnosed adult and pediatric patients will provide valuable information on the demographics, risk factors, evaluation, treatment, and outcomes of CTEPH in the United States.

## Abbreviations

BPA: balloon pulmonary angiography
CTA: computerized axial tomography angiography
CTEPH: chronic thromboembolic pulmonary hypertension
DSA: digital subtraction angiography
HRQoL: health related quality of life
MRA: magnetic resonance angiography
PAH: pulmonary arterial hypertension
PH: pulmonary hypertension
PTE: pulmonary thromboendarterectomy
SF-36v2: Short-Form 36 Questionnaire, version 2
UCSD: University of California San Diego
US-CTEPH-R: United States Chronic Thromboembolic Pulmonary Hypertension Registry
WHO: World Health Organization
